# Quantum Tunneling: History and Mystery of Large Amplitude Motions over a Century

**DOI:** 10.1021/acs.jpclett.4c02914

**Published:** 2024-12-20

**Authors:** Ha Vinh Lam Nguyen

**Affiliations:** † Univ Paris Est Creteil and Université Paris Cité, CNRS, LISA, F-94010 Créteil, France; ‡ Institut Universitaire de France (IUF), 1 rue Descartes, F-75231 Paris cedex 05, France

## Abstract

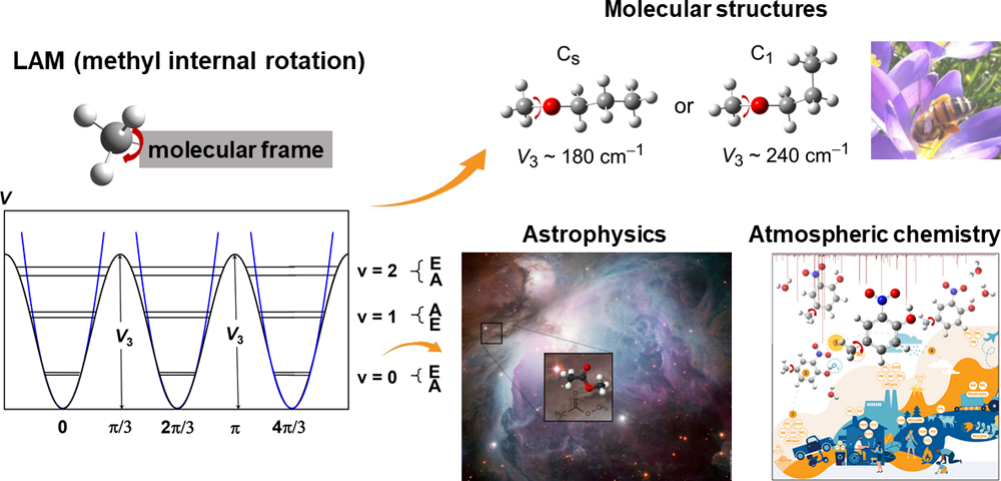

Large amplitude motions (LAMs), most notably represented by proton tunneling, mark a significant departure from small amplitude vibrations where protons merely oscillate around their equilibrium positions. These substantial displacements require tunneling through potential energy barriers, leading to splittings in, e.g., rotational spectra. Since Hund’s pioneering work in 1927, proton tunneling has offered a unique glimpse into the internal dynamics of gas-phase molecules, with microwave spectroscopy being the key technique for such investigations. The ubiquous LAM type is methyl internal rotation, characterized by 3-fold potentials arising from the interaction between methyl rotors and their molecular frame, with the barrier hindering methyl torsion and the orientation of the torsional axis being defining features. Investigating methyl internal rotations plays a key role in fields ranging from molecular physics, where the methyl rotor serves as a sensitive probe for molecular structures, to atmospheric chemistry and astrophysics, where methyl-containing species have been detected in the Earth’s atmosphere and interstellar environments and even discussed as potential probes for effects beyond the standard model of physics. Despite nearly a century of study, modeling methyl internal rotations with appropriate model Hamiltonians and fully understanding the origins of these motions, particularly the factors that influence torsional barriers, remain partially unresolved, reflecting the enduring mystery of quantum tunneling. This Perspective reviews the history of LAMs, highlights advances in decoding their complex spectra, and explores future research directions aimed at uncovering the remaining mysteries of these fascinating motions.

In classical mechanics, the concept of “tunneling” typically refers to the idea of constructing a tunnel through a mountain, allowing us to minimize the energy investment compared with the longer, more energy-intensive path of climbing over it. In quantum mechanics, tunneling is a fascinating phenomenon where particles, such as nuclei, traverse potential energy barriers in a process known as quantum tunneling without needing to “construct” a tunnel.

Almost a century has passed since Hund first discussed tunneling in polyatomic molecules as a path between “two mirror-image arrangements of lowest potential energy” in 1927,^1^ soon after the publication of Schrödinger’s equation.^2^ He referred to a double-well potential, using *inter alia* ammonia as an example, where the inversion tunneling motion involves the nitrogen atom passing through the plane spanned by the three hydrogen atoms (see Figure 1). Earlier and clear spectroscopic signatures of tunneling were mostly observed for systems in gas phase, with first observation indeed in the rotational spectrum of ammonia,^3^ but predominantly involves protons rather than heavier elements like nitrogen. Proton tunneling is the most commonly observed type of quantum tunneling due to the proton’s quantum nature and its small mass, which increases the tunneling probability. This probability is expressed as  where ω is the barrier width, *E* is the barrier height, *h* is Planck’s constant, and *m* is the mass of the tunneling particle.^4^ The barrier height and width vary drastically upon the environment surrounding the proton due to its much more localized nuclear wave function compared with, for example, electrons.

**Figure 1:**
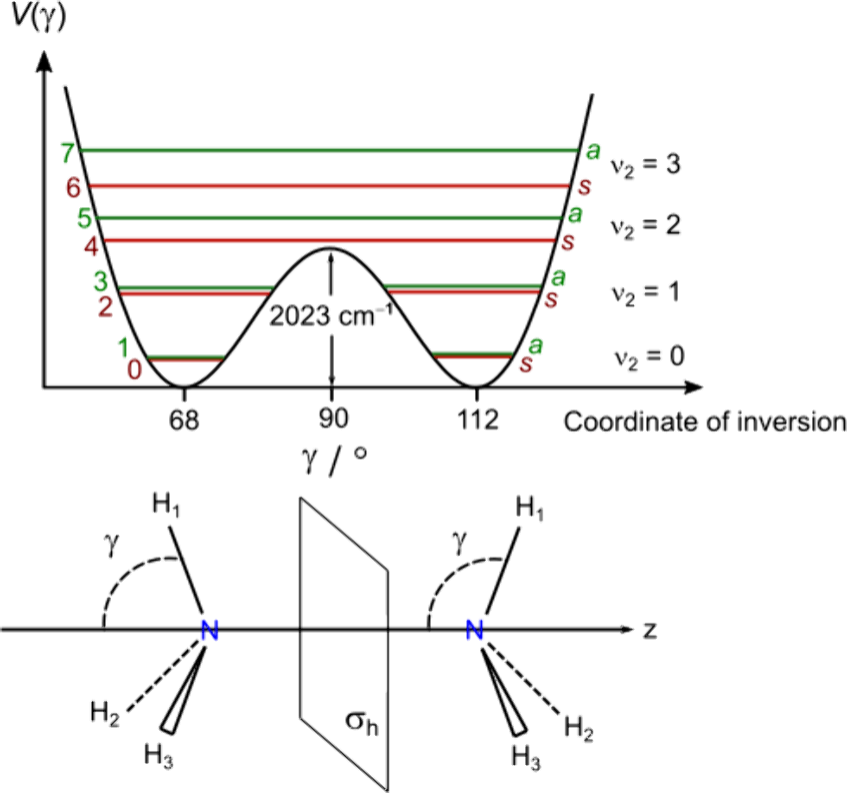
Tunneling LAM in ammonia. Upper trace: The 2-fold potential of NH_3_ along the inversion coordinate γ, showing the inversion vibration mode ν_2_ and the first excited energy levels labeled ν_2_ = 1, 2, and 3. Each subcomponent is either labeled *a* or *s*. An alternative labeling of the energy levels uses an ν_inv_ quantum number. Lower trace: the two equivalent equilibrium configurations of NH_3_, with the plane σ_h_ perpendicular to the *C*_3_ axis (the *z* axis).

Unlike small-amplitude vibrational motions, where protons oscillate much less than their bonding distance away from their equilibrium positions, tunneling is classified as large amplitude motion because the protons move about or even beyond their bonding distance away from their equilibrium structures. This motion causes splittings in the rotational spectra, can be effectively decoupled from other vibrational modes, and treated as a one-dimensional problem at low rotational temperatures. For example, in ammonia, the double minimum potential describes an inversion motion.^5^ In certain ring compounds, the ring puckering potential also exhibits a double-minimum form.^6^ Molecules with methyl group display a 3-fold potential, where the LAM is known as methyl internal rotation.^7^ The energy levels associated with these motions are calculated by solving one-dimensional wave equations, using appropriate reduced masses and potential functions, periodic for internal rotation and nonperiodic for inversion and ring puckering.

The study of potential functions and barrier heights is crucial for testing and refining model Hamiltonians and quantum chemical predictions, as it provides observational insights into the forces driving conformational preferences and thus the stability of rotational isomers. Microwave spectroscopy is particularly effective for investigating LAMs. This well-developed but not well-known analytical technique has been used extensively to determine the conformational structures of gas-phase molecules. The microwave spectra are highly specific to different conformers, thereby also enabling their detailed characterizations.^8^ Its unparalleled precision in determining barrier heights arises from the ability to resolve hyperfine details in rotational spectra, especially under supersonic jet conditions. Other spectroscopic techniques do not achieve the same level of accuracy. For example, infrared (IR) and Raman spectroscopy can probe vibrational motions associated with LAMs, but their resolution is limited, and their applicability in the gas phase is often constrained by signal-to-noise challenges.^9,10^ Time-resolved fluorescence spectroscopy has been used to study torsional or wagging motions, but it typically operates in condensed phases and provides less detailed insights into potential energy barriers.^11^ In condensed phases, nuclear magnetic resonance (NMR) spectroscopy and ultrafast techniques may capture dynamic information.^12^ However, these techniques are fundamentally different in scope and lack the direct measurement of quantum tunneling splittings that microwave spectroscopy excels at detecting. By directly measuring the rotational torsional transitions of isolated molecules, microwave spectroscopy provides a uniquely rigorous means of exploring the quantum nature of LAMs, making it the preferred technique for such studies.

Among the various types of LAMs, internal rotation has been the most extensively studied. This LAM is characterized by the presence of a moiety internally rotating relative to the rest of the molecule, known as the frame. The internal rotor can be asymmetric (for example NH_2_ or OH groups), but much more often symmetric (like a methyl group). The symmetry of both the rotor and the frame determines the number of equivalent minima in the torsional potential. For a methyl group with *C*_3_ symmetry attached to an asymmetric *C*_1_ frame, a 3-fold potential arises, as described in Figure 2 drawn according to the following equation:^8^
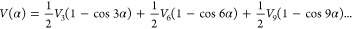
1

**Figure 2:**
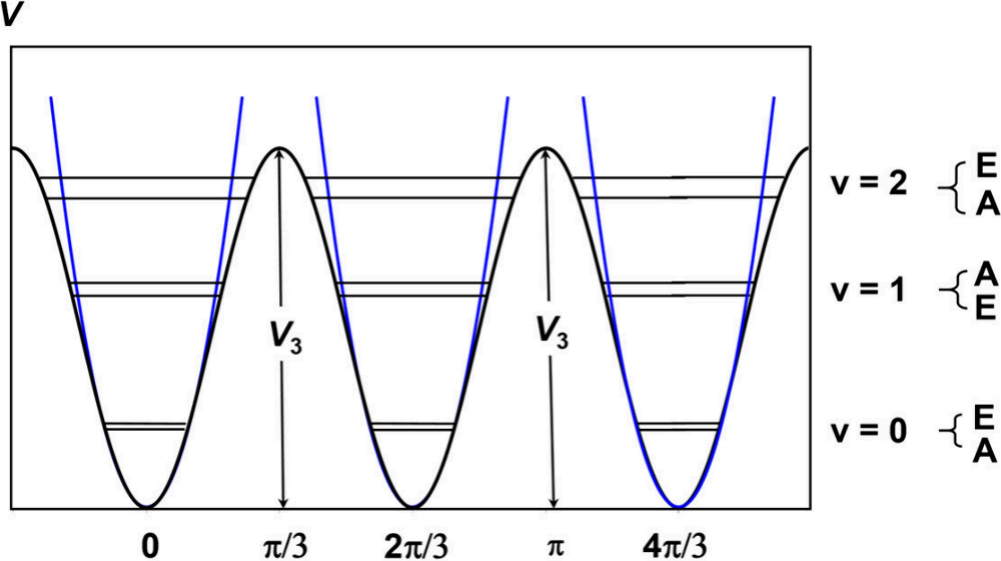
A schematic illustration of the potential function and torsional energy levels for a 3-fold internal rotor. Each torsional energy level is identified by quantum number *v*. The torsional sublevels are labeled as A or E under the *C*_3_ symmetry group. If the barrier height is infinite, the internal rotation can be described as three harmonic oscillators (shown in blue), resulting in 3-fold degenerate sublevels.

When a single methyl internal rotor is present, each rotational state splits into two symmetry species, A (nondegenerate) and E (2-fold degenerate), as shown in Figure 2. In the rotational spectrum, these A–E splittings depend on the potential barrier hindering the methyl torsion. The barrier height can vary widely from nearly free rotation (close to 0 cm^–1^) to over 1000 cm^–1^. If the barrier height exceeds 1000 cm^–1^, the methyl protons’ motions can be described as those of three harmonic oscillators with degenerate energies, i.e., no resolvable A–E splittings occur, such as the molecules can be described as semirigid rotors. Their microwave spectra can be accurately modeled using a rigid-rotor Hamiltonian with centrifugal distortion corrections, as demonstrated by numerous high-resolution rotational spectroscopic studies.^13,14^ Conversely, if the barrier is negligible, the system approaches the limit of free internal rotation, as seen for example in the methyl group of CH_3_–C≡C–CD_3_.^15^ For many molecules that fall between the extremes of harmonic oscillation and free internal rotation, resolvable A–E splittings of different orders of magnitude are observed.^16−20^

Multiple methyl internal rotors lead to much more complex spectral patterns. The number of torsional species and the torsional splittings depend on the number of methyl rotors, their barriers, their interactions, and frame symmetry. For example, in a two-top molecule with nonequivalent methyl groups, the fine structure consists of quintets, as shown in Figure 3. The torsional species are labeled as (00), (01), (10), (11), and (12) with three torsional states for each methyl top denoted as σ = 0, 1, 2. Investigations of two-top molecules are still rather scarce. Some examples are methyl propionate,^21^ the series of dimethylfluorobenzene,^22,23^ dimethylthiazole,^24,25^ dimethylanisole,^26,27^ and dimethylbenzaldehyde.^28^ If the methyl groups are equivalent, quartets are observed since the (01) and (10) levels become degenerate (see Figure 3). Due to the additional molecular symmetry requirement of the frame, e.g., *C*_2_, *C*_2h_, or *C*_2v_, the number of studies reported in the literature are even smaller, with acetone^29,30^ and dimethyl ether^31^ as two prototypical examples. Even fewer studies have been conducted on molecules with three methyl internal rotors such as trimethylsilyl iodide, (CH_3_)_3_SiI^32^ and similar systems^33^ and *N*,*N*-dimethylacetamide,^34^ and only one molecule with four methyl rotors, 2,3,4,5-tetramethylthiophene, has been reported in an ongoing study.^7,35^

**Figure 3:**
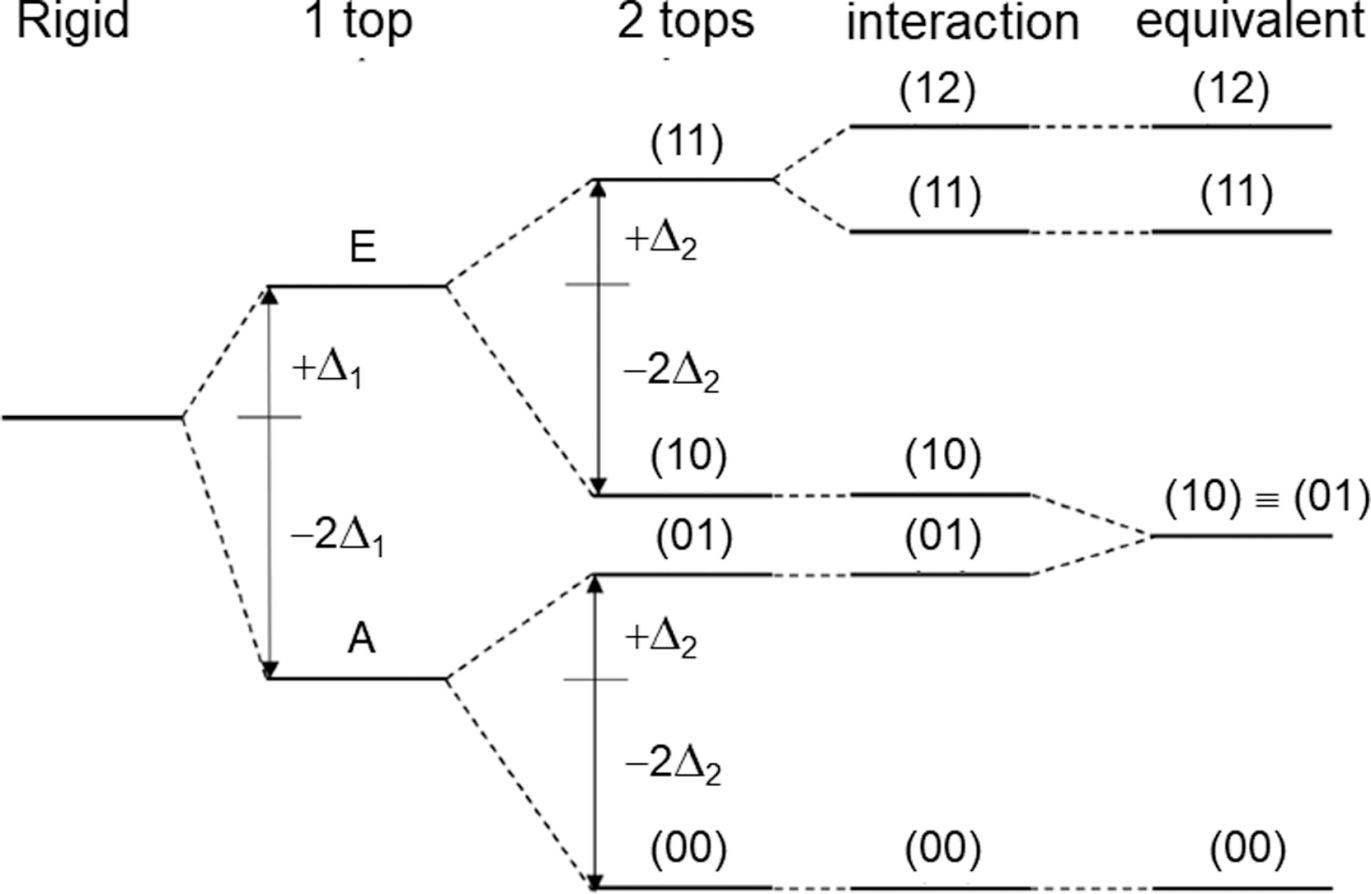
Energy correlation diagram for molecules without feasible methyl internal rotation, with feasible torsion due to one methyl top (A–E), with feasible simultaneous torsions of two nonequivalent tops, and with feasible simultaneous torsions of two equivalent tops using the semidirect notation (σ_1_,σ_2_).

**Why** is **internal rotation** significant, and why should we focus on analyzing these spectra in the laboratory? One major reason is that it helps us understand the ***molecular structure***, which in turn can shed light on (bio)chemical properties. The torsional barriers of the methyl tops can offer insights into functional groups and structural features, effectively making the methyl group a “spectroscopic probe” for determining local properties of molecular structure. This approach is especially valuable when studying larger, more complex biomolecules or natural substances. A study of saturated methyl alkyl ketones, compounds naturally present in honey bee pheromones, revealed that molecules with *C*_*s*_ symmetry consistently exhibit a barrier to methyl internal rotation around 180 cm^–1^, while those with *C*_1_ symmetry display a barrier close to 240 cm^–1^, as shown in Figure 4.^36^ The molecular geometries of pheromones provide valuable insights that help address intriguing questions like: How do these molecular shapes interact with insect receptors? Can the structural information on pheromones reveal details about the binding sites on olfactory membranes?

**Figure 4:**
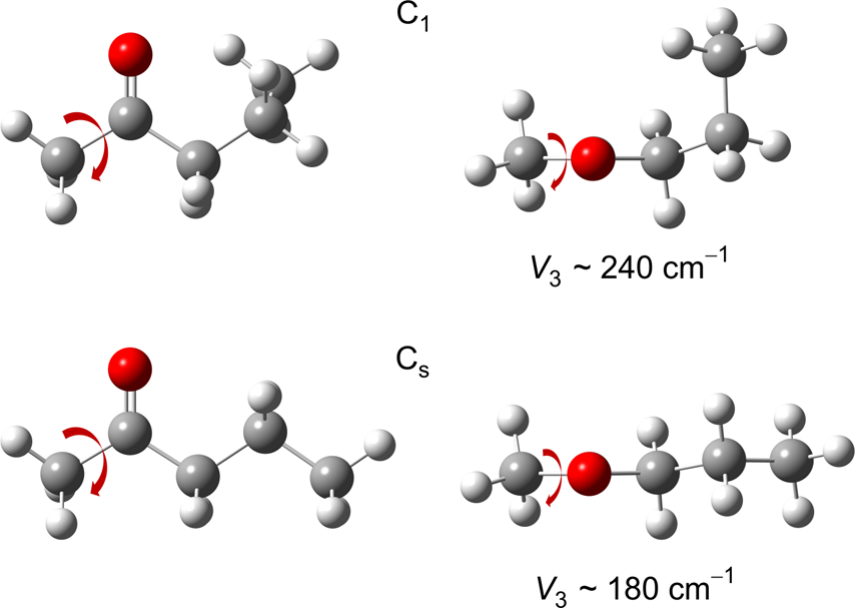
Two conformers of pentan-2-one, a honey bee pheromone compound, illustrate how the barrier to methyl internal rotation relates to the molecular structure. The *C*_*s*_ conformer, where all heavy atoms lie on a symmetry plane, has a barrier of 180 cm^–1^. In contrast, the *C*_1_ conformer, with the γ methyl group tilted out of the C–(C=O)–C plane, exhibits a higher barrier of around 240 cm^–1^.^37^

In conformational analysis using internal rotation, lavender oil’s main component, linalool, is a compelling example. As an acyclic monoterpene, the large size and open structure of linalool give it a vast conformational landscape with hundreds of plausible conformers. Yet, despite these possibilities, only one conformer was detected in the jet-cooled microwave spectrum. As low-energy conformers yield very similar calculated rotational constants, the decisive factor in identifying the observed conformer was the angle between the methyl rotor axis and the principal axes, which only matched with a single conformer (see Figure 5).^38^ Thus, analyzing internal rotation is vital for determining the structures of larger molecules.

**Figure 5:**
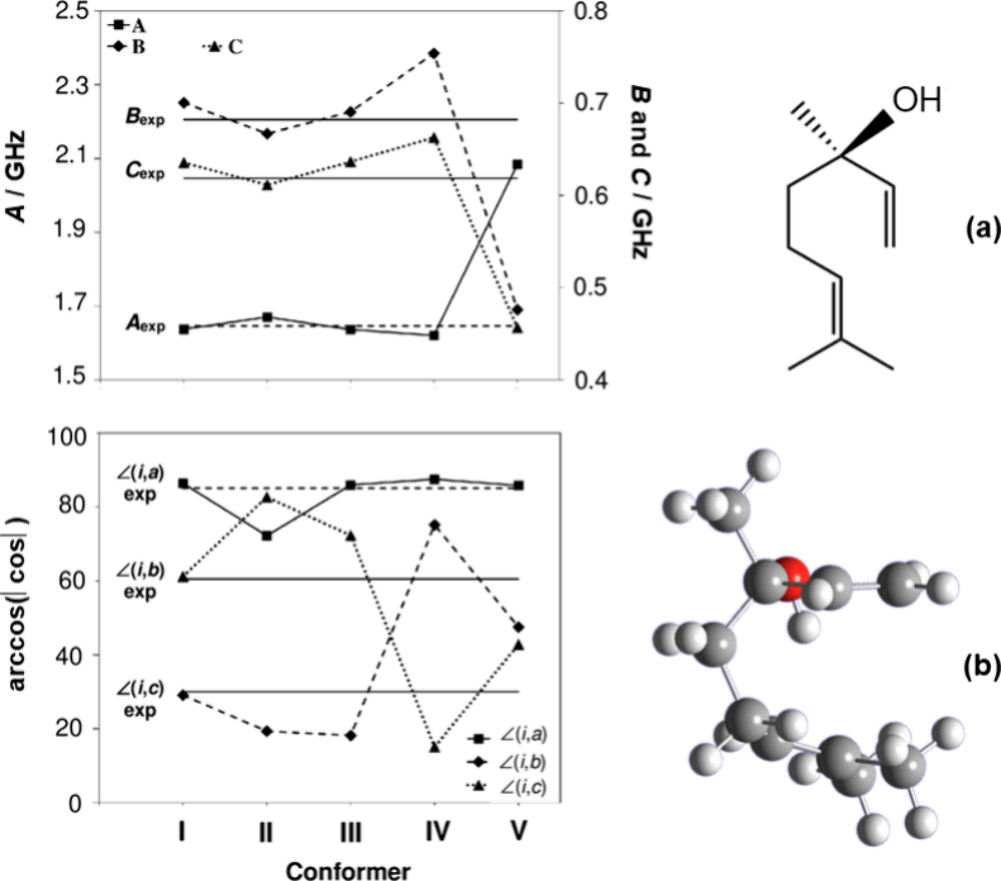
Comparison of the rotational constants *A*, *B*, *C* and angle between the methyl rotor axis *i* and the principal axes *a*, *b*, *c* between theory (dots) and experiment (exp, horizontal lines) to assign the observed conformer (b) of linalool (a).^38^

When discussing natural substances or biological molecules, the influence of water molecules in biological environments cannot be overlooked as they play a pivotal role in shaping molecular properties and interactions. Water complexation often complicates the potential energy landscape and dynamics of internal rotation. The effect of water on methyl internal rotation is sometimes negligible, but it can also significantly alter or even quench the proton tunneling due to the formation of intramolecular van der Waals bonds with a hydrogen atom of the methyl group or through structural changes induced by water. In 4-hydroxy-2-butanone, water dramatically influences the molecular structure. To accommodate water molecules, the heavy-atom skeleton of 4-hydroxy-2-butanone undergoes significant reshaping, deviating from the geometry of the free monomer.^39,40^ The methyl internal rotation is also altered upon water complexation and structural adaptation of the molecule (Figure 6). While for the monomer the methyl torsional barrier is 209 cm^–1^, the addition of a water molecule increases this barrier to 282 cm^–1^. Adding more water molecule quenches the methyl internal rotation entirely. For all complexes with 2–5 water molecules, the A–E splittings become too small to be resolved. These findings provides a detailed molecular description of how molecules dynamically adapt to water. A similar pattern is observed in methyl carbamate, a biologically active molecules due to its resemblance to peptide bonds. The methyl torsional barrier of the monomer is 352 cm^–1^,^41^ which increases progressively to 374 cm^–1^, 386 cm^–1^, and 405 cm^–1^ with the successive addition of 1, 2, and 3 water molecules.^42^ These results underscore the profound impact of water on the torsional dynamics of methyl groups and the structural flexibility of biologically relevant molecules.

**Figure 6:**
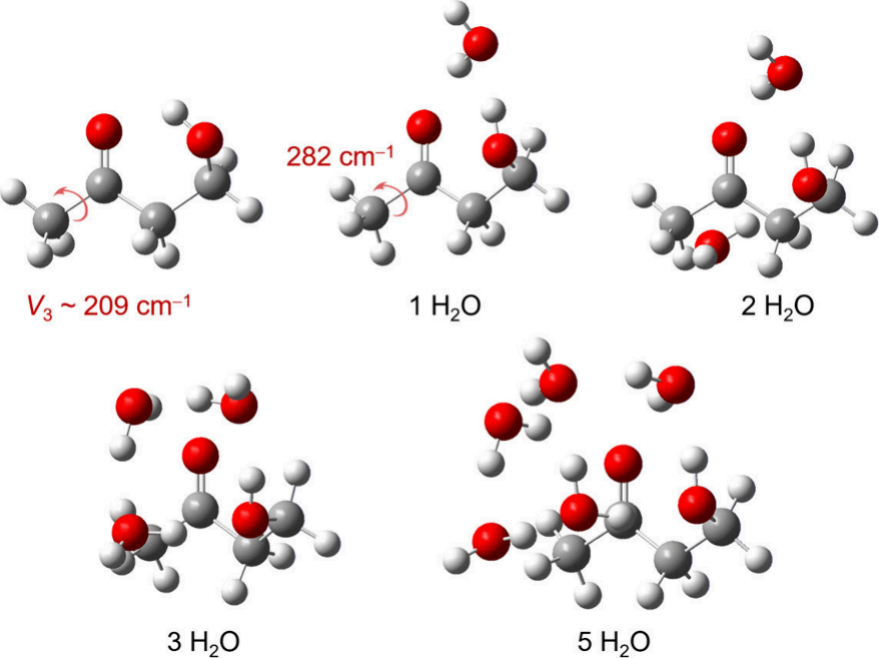
Structural adaptation and changes in methyl internal rotation barriers in 4-hydroxy-2-butanone upon water complexation. To accommodate water molecules, the heavy-atom skeleton undergoes notable reshaping, diverging significantly from the geometry of the free monomer. The methyl torsional barrier increases from approximately 209 cm^–1^ in the monomer to 282 cm^–1^ in the monowater complex. With further water molecules, the methyl internal rotation is entirely suppressed.^39,40^

Chirality is a fundamental aspect of biologically active molecules. Nearly all essential bioactive compounds are chiral, and typically, only one enantiomer exhibits the intended biological activity, pharmacodynamics, and toxicity. The chemistry of life is characterized by “homochirality”, relying almost exclusively on left-handed amino acids and right-handed sugars. This unique handedness is critical for the structure and function of biomolecules, enabling precise interactions in biological systems. Pharmaceuticals are often chiral, because only the correct enantiomer can perform an effective “handshake” with receptor sites. Consequently, half of the top 100 drugs are sold as single enantiomers, and the majority of drugs entering the development pipeline are enantiomerically pure. Tunneling between enantiomers in biologically relevant molecules is generally suppressed due to the high energy barriers separating the two forms. For example, flurbiprofen, a chiral molecule, exhibits a theoretical double-well potential for conformational transitions. However, the barrier is typically too high for feasible tunneling under standard conditions.^43^ On the other hand, some molecules exhibit transient chirality or atropisomerism, where barrier height significantly influences the equilibrium population of pseudoenantiomers. Benzyl alcohol and benzyl mercaptan are examples. For benzyl alcohol, the concerted rotation of the CH_2_OH and OH groups above the phenyl ring constitutes a tunneling motion, with a barrier of approximately 280 cm^–1^ that interconverts enantiomers (see Figure 7).^44^ Similarly, benzyl mercaptan features a slightly lower barrier of around 248 cm^–1^ due to the inversion of the CH_2_SH and SH groups. The spectrum is characterized by torsional tunneling doublings, strongly perturbed by Coriolis interactions.^45^ Recently, the microwave six-wave mixing approach was applied to induce transient enantiomeric excess in a quantum racemic mixture of benzyl alcohol, showcasing the potential to manipulate chirality.^46^

**Figure 7:**
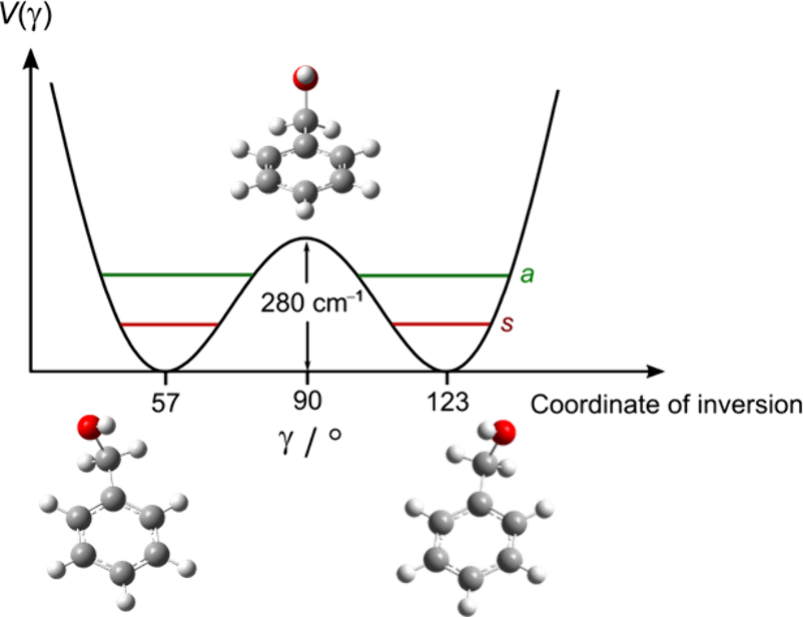
2-fold potential of benzyl alcohol along the CH_2_OH inversion coordinate γ, showing the vibrational ground state with its subcomponents *a* and *s*. The tunneling motion with a barrier of approximately 280 cm^–1^ interconverts the two enantiomers.

The first internal rotors studied by microwave spectroscopy were often molecules identified in ***astrophysical*** environments. Consequently, the history of internal rotation studies is closely tied to astrophysical discoveries. Understanding internal rotation is crucial for detecting molecules in the interstellar medium, where over 300 molecules have been identified in circumstellar shells or interstellar clouds. Sixteen of these molecules exhibit methyl internal rotation: CH_3_OH, CH_3_SH, CH_3_CHO, CH_3_NH_2_, CH_3_NCO, HC(O)OCH_3_, CH_3_COOH, CH_3_CHNH, CH_3_OCH_3_, CH_3_CONH_2_, CH_3_NHCHO (tentative), CH_3_COCH_3_, CH_3_CHCH_2_O, CH_3_OCH_2_OH, CH_3_COOCH_3_, C_2_H_5_OCH_3_.^47^ A well-known example is methanol with a methyl internal rotation barrier of approximately 380 cm^–1^, detected in 1976 in Orion-A.^48^ Churchwell and Winnerwisser discovered methyl formate around the same time in Sgr B2, recognizing the A–E doublet of the 1_10_ ← 1_11_ transition.^49^ Acetaldehyde was later found in Sgr B2,^50^ as well as in TMC-1 and L134N,^51^ while acetic acid was first identified in the interstellar medium by Mehringer et al.^52^ Other molecules containing internal rotors, such as acetone,^53^ dimethyl ether,^54^ and ethyl methyl ether^55^ have also been observed. A noteworthy discovery was made in 2013 when methyl acetate, which has five torsional fine components due to two nonequivalent methyl rotors, was detected in the Orion cloud.^56^ These detections were made possible through the collaboration between laboratory studies and interstellar surveys conducted in the microwave, millimeter, and submillimeter wave regions.

Astrophysical observation of LAMs, in both forms of inversion and internal rotation, has proven invaluable for exploring possible changes in fundamental constants, particularly the proton-to-electron mass ratio, μ = *m*_*p*_/*m*_*e*_ ≈ 1836, from the early Universe to the present day.^57^ The hypothesis of time- or space-varying coupling constants arises from the fact that the fundamental forces of nature are not fixed by the standard model of physics, leaving room for variations. The sensitivity of molecular transitions to such variations provides a unique method to investigate the stability of the laws governing the cosmos on vast time scales. Any drift in constants results in frequency shifts Δν for specific transitions, which are, to a first order approximation, proportional to the fractional change in μ. Methanol is particularly suitable for such studies due to the coupling between its methyl internal and the overall rotations, which enhanced the sensitivity for a possible drift in μ in specific transitions.^58,59^ The degree of sensitivity is quantified by the *K*_*μ*_ coefficients defined as Δν/ν = *K*_*μ*_ · Δμ/μ. The broad range of *K*_*μ*_ values for methanol transitions enables robust constraints on μ using a single molecular species, reducing uncertainties arising from chemical segregation. Using measurements of ten different absorption lines spanning a wide range of *K*_*μ*_ values and a potential spatial differentiation of the E- and A-symmetry species in the gravitationally lensed galaxy PKS1830–211 (observed with three different radio telescopes), the μ variation has been determined to be Δμ/μ = (1.5 ± 1.5) × 10^–7^ with a purely statistical 1σ constraint at the redshift *z* = 0.89. This corresponds to a lookback time of 7.5 billion years - more than half Universe’s age.^60^ Accounting for systematic uncertainties refined this estimate to Δμ/μ = (−1.0 ± 0.8_stat_ ± 1.0_sys_) × 10^–7^.^61^ This translates to a maximum variation rate of 2 × 10^–17^ per year, matching the precision achieved in laboratory experiments with the best optical clocks.^62^ These findings strongly suggest that fundamental constants have remained extraordinarily stable over cosmological time scales, with methanol emerging as the most sensitive molecular probe for time variations of μ. Ammonia provides another valuable system due to its inversion transitions, which are sensitive to changes in μ with *K*_*μ*_ = −4.45, also showcasing the versatility of LAMs as probes of fundamental physics across the Universe’s history. Observations of ammonia absorption lines have resulted in 1σ constraints of Δμ/μ = (1.0 ± 4.7) × 10^–7^ for the object PKS1830–211 at *z* = 0.89^63^ and (−3.5 ± 1.2) × 10^–7^ for the object B0218 + 357 at *z* = 0.68.^64^

In addition to their significance in astrophysics, molecules with methyl internal rotation are crucial in ***atmospheric chemistry***. With increasing sensitivity for atmospheric detection, molecules exhibiting internal rotors are observed more frequently. They are assumed to play an important role in the chemistry of the Earth’s atmosphere. Even though present only in trace amounts as volatile organic compounds (VOCs), these substances are major contributors to atmospheric reactions. For example, methyl-substituted compounds such as isoprene and other terpenes, emitted by plants, contribute to the formation of secondary organic aerosols (SOAs) and ozone, impacting air quality and climate. Methylated oxygenated aromatic compounds, such as phenol and nitrophenol derivatives, form a significant class of pollutants and aerosol precursors released into the atmosphere by combustion. Their reactions increase the levels of oxidants such as OH radicals and promote the formation of SOAs. Despite their relevance to the Earth’s atmosphere, significant gaps in our understanding of how these compounds interact with other atmospheric molecules, particularly water, remain, highlighting the need for detailed characterization of their molecular structures. Methyl internal rotation provides insights into the molecular structures of not only isolated molecules but also their complexes with water, which is vital for understanding their evolution from early aggregation stages and cluster growth to aerosol formation.

A classic example of the influence of water on methyl torsional barriers is the acetone–water complex. Acetone is a prevalent carbonyl compound in the Earth’s atmosphere, acting as a significant contributor to the generation of OH radicals through photolysis, which makes it a key player in atmospheric chemistry processes. Interactions with water disrupt the symmetry of two methyl groups in acetone, leading to a significantly lower torsional barrier for the methyl group nearest the water molecule compared to that of isolated acetone. The barrier for the more distant methyl group is also reduced, though to a lesser extent.^65^ Staying in the ketone family, the trend that the *V*_3_ values decline upon complexation is also observed for the four water-bound conformers of methyl vinyl ketone, an atmospheric oxidation product,^66^ as well as on acetophenone,^67^ likely due to electron density depletion at the oxygen atom caused by water binding. Atmospheric oxygenated organic compounds, formed from the photo-oxidation of VOCs, further illustrate these effects. For example, *p*-toluic acid, a photo-oxidation product of *p*-xylene, and pyruvic acid, a major isoprene oxidation product, display diverse responses to water complexation. The methyl torsional barriers sometimes increase slightly, as with pyruvic acid,^68^ or remain largely unchanged, as seen in *p*-toluic acid, which exhibits a very low barrier.^69^ It is important to note that unlike biologically relevant molecules, most atmospherically significant species are smaller, allowing water interactions to exert a more pronounced influence on their internal dynamics. This variability underscores the nuanced effects of water on methyl internal rotations, depending on the molecular structure and the nature of the interactions.

Understanding LAMs has always been a mystery in the context of methyl internal rotation. The microwave spectrum of such molecules typically shows splittings in each rotational transition originating from torsional sublevels, which can no longer be analyzed with the common rigid rotor Hamiltonian (**H**_**r**_) supplemented with centrifugal distortion (**H**_**CD**_) corrections. When the internal rotation cannot be modeled as a small amplitude motion, the analysis becomes significantly more difficult and perturbation methods often fail. Despite considerable advancements over the last decades, only a handful of **computational programs**, such as *XIAM*,^70^*BELGI*,^71^*RAM36*,^72^*ERHAM*,^73^*aixPAM*,^74^*ntop*,^26^ and *westerfit*(75) have been developed to address the effects of internal rotation. When the barrier to internal rotation is particularly low, assigning spectral lines can be a daunting task, often requiring individual treatment of different symmetry species and, if possible, extensive use of combination difference loops to confirm the correctness of the assignment. It has been shown that treating the A species as a semirigid rotor with centrifugal distortion corrections (**H** = **H**_**r**_ + **H**_**CD**_) produces fits with standard deviations close to experimental accuracy, even in cases of very low methyl rotation barriers.^7^ By adding odd power effective Hamiltonian terms (**H**_**op**_), as implemented in LAM programs like *SFLAMS*,^76^*WS18*,^77^ and *spfit*,^78^ it is possible to achieve deviations as low as the experimental accuracy when individually fitting the torsional excited states (E species, (01), (10), (11), (12) etc.).^7,22−24^

Theoretical and computational approaches have provided profound insights into quantum tunneling and barrier crossing in chemical reactions, offering valuable perspectives on LAMs. Methods such as instanton theory, path integral approaches, and extensions of transition state theory that account for quantum effects have been successfully applied in diverse contexts, including enzymatic reactions and astrochemical processes, where they effectively model tunneling rates and dynamics on complex potential energy landscapes.^79^ Computational chemistry has further explored tunneling in various scenarios, such as its role in catalysis and kinetics^80^ as well as in elucidating reaction mechanisms under extreme conditions or within enzymes.^81,82^ Quantum chemical studies also offer critical insights into the interplay of quantum effects with molecular rotations and vibrations. In gas-phase microwave spectroscopy, these calculations are especially important for studying LAMs. They provide theoretical predictions for molecular parameters, such as initial values and potential energy features, which guide spectral assignments and validate experimental observations.^83^ Since quantum chemical calculations are typically performed on isolated molecules, they align closely with the conditions of gas-phase spectroscopy, enabling highly accurate comparisons.

Through the long history of LAMs and microwave spectroscopy, the number of investigated molecules exhibiting internal rotation effects has significantly increased in recent decades. Among the various types of molecules with LAMs, those with **conjugated double bonds** stand out due to their ability to transfer information on structures and internal dynamics across longer molecular distances via π-electrons.^84^ This allows interactions between the LAMs and distant parts of the molecule. Notable studies have focused on benzene derivatives and five-membered aromatic rings. These investigations have demonstrated that steric hindrance from nearby substituents increases the methyl torsional barrier. For example, when a fluorine atom is adjacent to a methyl group, the barrier is around 220 cm^–1^, as seen in 2-fluorotoluene (molecule (**1**) in Figure 8) and its derivatives.^85,86^ Substituting fluorine with larger halogen atoms like chlorine, bromine, or iodine leads to a progressive increase in the barrier (469 cm^–1^, 502 cm^–1^, and 530 cm^–1^, respectively), in correlation with the size of the neighboring atom.^87^ A methoxy group, as found in *o*-methylanisole (**3**)^88^ and its derivatives,^89^ results in barriers around 444 cm^–1^. While steric effects as such are predictable, electronic interactions can sometimes cause unexpectedly low torsional barriers in sterically affected methyl rotors. Surprisingly low barrier heights are observed in cases such as the two methyl groups in 4,5-dimethylthiazole (**6**)^25^ or the 2-methyl group in 2,3-dimethylanisole (**5**)^90^ and 2,3-dimethylfuran (**7**),^91^ where electrostatic effects may contribute to reducing the barrier, despite significant steric obstruction.

**Figure 8:**
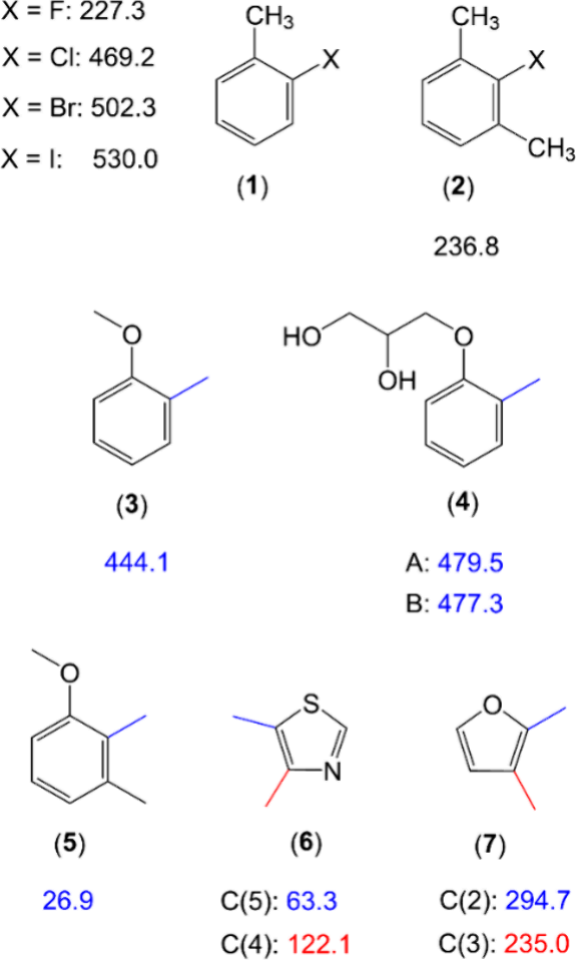
Different experimental methyl internal rotation barriers (in cm^–1^) in molecules with conjugated π-bonds: (**1**) 2-halotoluene,^85,87^ (**2**) 2,6-dimethylfluorobenzene,^86^ (**3**) *o*-methylanisole,^88^ (**4**) mephenesin (conformers A and B),^89^ (**5**) 2,3-dimethylanisole,^90^ (**6**) 4,5-dimethylthiazole,^25^ and (**7**) 2,3-dimethylfuran.^91^ Methyl rotors are color-coded for the sake of clarity. Barriers for the black methyl groups in molecules (**3**) and (**5**) are not included in the comparison and are therefore not provided.

**Why do the barriers to internal rotation differ** among seemingly similar methyl groups? Numerous explanations have been proposed, including electrostatic models, hyperconjugation, steric repulsions, and other concepts, but particular origins of these barriers remain an open debate, even after a century of theoretical and experimental work. The question of torsional potentials, even in the case of simple molecules such as ethane, continues to be a topic of ongoing discussion. This enduring mystery has been likened to the Bermuda Triangle of electronic structural theory.^92^ A thorough understanding of these barriers requires breaking them down into components such as hyperconjugation, Pauli exchange steric repulsion, and relaxation effects, particularly the changes in bond and lone-pair energies. Many efforts have been made to explain these barriers by isolating one or two of the contributing factors. However, the key idea presented here is that the mechanisms driving methyl torsional barriers become clear only when the combined effects of all of the major energetic interactions involved in the rotation process are considered. The barrier can also be analyzed through energy decomposition into kinetic and electrostatic potential energy components, such as electron–nuclear repulsion and electron–nuclear attraction.

A common misconception that has obscured our understanding of the torsional barrier mechanisms is the assumption that steric repulsion increases the barrier when molecular moieties are brought into closer proximity during rotation. As mentioned above, in molecules with π-electron systems such as aromatic compounds, the interactions between steric and electrostatic forces become even more complex. In these systems, steric hindrance can distort the conjugated π-cloud, affecting the electronic distribution and influencing the torsional barrier. Additionally, electrostatic interactions between π-electrons and neighboring atoms or groups can further complicate the energetic landscape, sometimes lowering the barriers of evidently sterically hindered methyl rotors.^25,84,90,91^ This interplay between steric and electrostatic effects highlights the need to consider both factors when analyzing such systems as they can significantly alter molecular behavior. A noteworthy example of this is the “*cis* effect” on methyl internal rotation in 1-substituted propene derivatives,^93^ four such molecules are shown in Figure 9. In all cases, the Z isomer exhibits greater stability than the E isomer due to an attractive intramolecular nonbonded electrostatic interaction between the substituent and the positively charged in-plane hydrogen atom of the methyl group. This is supported by the charge distribution depicted in Figure 9. Such interactions are unusual, because they typically occur only when the distance between the interacting atoms is less than the sum of the van der Waals radii, a condition met by all four molecules. Therefore, the Z isomer has a unique feature: one hydrogen atom of the methyl group is eclipsed by the substituent at position 1 in the equilibrium structure. This configuration results in a radically lower methyl torsional barrier than that of the E form. If only steric hindrance were considered, the Z isomer would be expected to have a barrier higher than that of the E isomer, but the contrary is observed. The nonbonded repulsion between the 1-substituent and the methyl group pushes the in-plane hydrogen atom away from the substituent, leading to a reduction in the methyl torsional barrier. Differences in the barrier heights among the four molecules likely result from a combination of factors, including the strength of electrostatic attraction (primarily determined by the charge distribution), the nonbonded distances, and the van der Waals radii.

**Figure 9:**
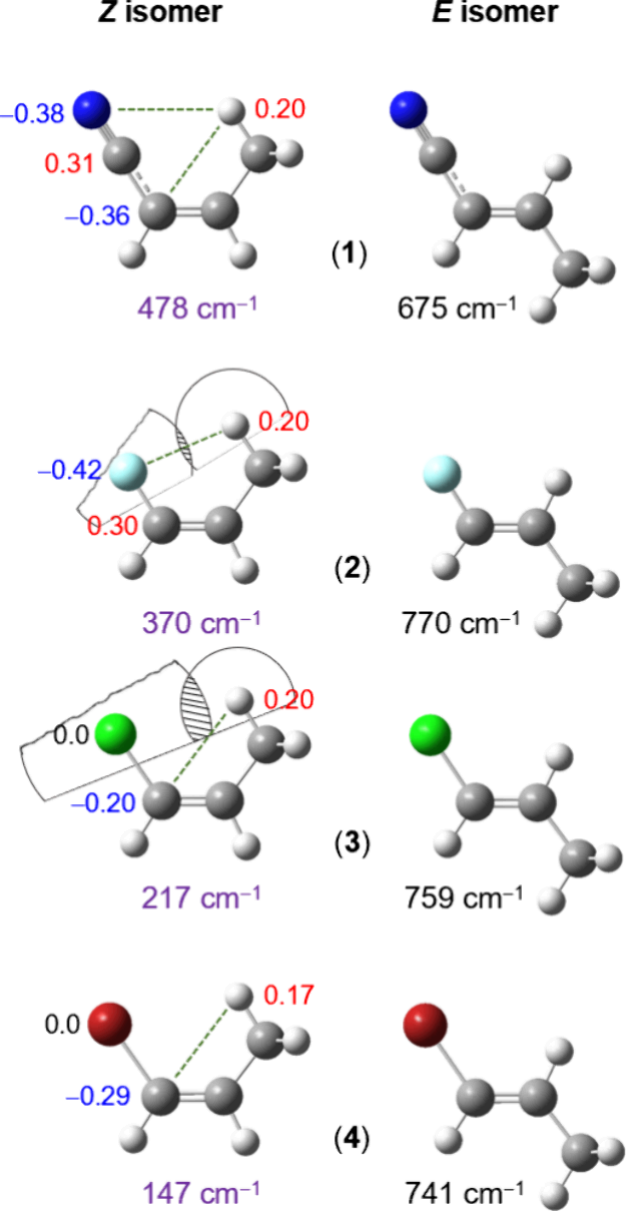
Experimental barriers to methyl internal rotation in 1-substituted propene derivatives, with values for E isomers in black and for Z isomers in lila. (**1**) 1-cyanopropene,^93^ (**2**) 1-fluoropropene,^94,95^ (**3**) 1-chloropropene,^96,97^ and (**4**) 1-bromopropene.^98^ Partial charges at the Z isomers, derived from NBO calculations, are displayed in blue and red. Potential nonbonded electrostatic interactions are depicted by dotted green lines.

For all LAMs, the most defining feature is the barrier that restricts the motion with larger splittings generally observed for lower barriers. Predicting these barrier heights remains challenging, as chemical intuition often falls short and quantum chemical methods are still not accurate enough for the subtle balance of multiple effects. Both steric and electrostatic effects play critical roles, especially in the case of methyl rotors experiencing π-electron conjugation, which can propagate effects over longer molecular distances. Over the past century, our understanding of LAMs, notably methyl internal rotations, has advanced significantly, paralleling broader progress in physical chemistry. The history of LAMs has revealed many mysteries, as much of their fascinating behavior remains unexplained despite the significant progress that we have made. Even after a century of study, we are still in the early stages of quantitatively understanding large amplitude motions. There is no doubt that many future investigations into LAMs will be undertaken to unveil the remaining mystery. A wealth of information awaits: from detecting new molecular species in space and the Earth’s atmosphere, determining the presence of extraterrestrial chemistry and trace pollutants, respectively, and revealing the precise structures of complex molecular systems.
